# Safety and Efficacy of Therapeutic Cancer Vaccines Alone or in Combination With Immune Checkpoint Inhibitors in Cancer Treatment

**DOI:** 10.3389/fphar.2019.01184

**Published:** 2019-10-11

**Authors:** Jing Zhao, Ye Chen, Zhen-Yu Ding, Ji-Yan Liu

**Affiliations:** ^1^Department of Biotherapy, Cancer Center, and National Clinical Research Center for Geriatrics, West China Hospital of Sichuan University, Chengdu, China; ^2^Sichuan Clinical Research Center of Biotherapy, West China Hospital of Sichuan University, Chengdu, China

**Keywords:** cancer vaccine, immune checkpoint inhibitor, combination therapy, neoantigen, immunotherapy

## Abstract

Therapeutic cancer vaccines have proven to seldom induce dramatic clinical response when used alone, and therefore, they are being studied in combination with additional treatment modalities to achieve optimal treatment activities. Growing preclinical data show that combining vaccines and immune checkpoint inhibitors (ICIs) can prime intensified immunogenicity and modulate immunosuppressive tumor microenvironment. Herein, we focus on the safety and efficacy of approved and promising cancer vaccines alone or combined with ICIs in the treatment of several malignancies. Generally, the majority of clinical trials support the concept of synergy that combination therapy of vaccines and ICIs holds maximized potential to improve clinical outcomes. Importantly, the combination has acceptable safety and minimal additional toxicity compared with single-agent vaccines or ICIs. Additionally, the potential strategies of combining personalized tumor vaccines with ICIs will become priority option and future direction of vaccine development and application and the urgent need to develop effective biomarkers to screen appropriate patient populations and predict response to combination therapy.

## Introduction

Cancer immunotherapy, including cancer vaccines, immune checkpoint inhibitors (ICIs), and adoptive cell therapy, represents a scientific breakthrough in the treatment of various malignancies (Kirkwood et al., 2012). Cancer vaccines are designed to specifically target tumor antigens and provoke host immune system to selectively fight against cancer cells (Melief et al., 2015). Currently, multiple cancer vaccine platforms have been developed, including peptide- or protein-based vaccines, oncolytic virus– or recombinant virus–vectored vaccines, dendritic cell (DC) vaccines, engineered cellular vaccines, and idiotype vaccines (Schlom, 2012; Guo et al., 2013). Generally, the majority of vaccines are well tolerated and have limited toxicity (Gatti-Mays et al., 2017). Unfortunately, with the recent failure of phase III clinical trial, vaccines as monotherapy have been shown to produce only modest or negative survival benefits (Hu et al., 2018; Gulley et al., 2019). Hence, combining therapeutic cancer vaccines with additional treatment modalities has been explored, as an approach to augment immune responses and treatment activities.

Malignant tumors may evade immune surveillance by utilizing inhibitory immunoregulatory mechanisms, especially immune checkpoint receptor pathways. ICIs can enhance antitumor immune response by blocking these negative regulation signaling and have revolutionized the treatment landscapes of different tumor types such as melanoma, lung, renal cell, and bladder cancers (Hodi et al., 2010; Topalian et al., 2012). Nonetheless, ICIs do not appear to achieve clinical improvement in some other malignancies, for example, prostatic and pancreatic cancers, and less than 20% of unselected patient response to single-agent ICI (Royal et al., 2010; Beer et al., 2016; Strauss et al., 2016). In addition, ICI therapy also induces inflammatory responses and toxicity referred to as immune-related adverse events (irAEs), which may affect multiple organs and range from mild and manageable to life-threatening (Champiat et al., 2015; Puzanov et al., 2017).

Recently, growing preclinical and clinical researches have tended to combining therapeutic cancer vaccines and ICIs to explore the synergistic effects. Herein, we focus on the safety and efficacy of approved (sipuleucel-T and talimogene laherparepvec [T-VEC]) and promising cancer vaccines alone or combined with ICIs (cytotoxic T-lymphocyte-associated protein 4 [CTLA-4] and, programmed cell death 1 [PD-1] and its ligands [PD-L1]) for the treatment of several malignancies. We highlight the enormous potential of personalized cancer vaccines in combination with ICIs, which can produce complete tumor regression in several studies, and hope to provide theoretical foundations and innovative ideas for the development and application of cancer vaccines in clinical settings.

## Therapeutic Cancer Vaccines

Currently, the Food and Drug Administration (FDA) has approved two therapeutic cancer vaccines: sipuleucel-T for metastatic castration-resistant prostate cancer (mCRPC) based on modest improvement in overall survival (OS), and T-VEC for unresectable advanced melanoma based on partial improvement in OS and durable response rate (DRR) (Kantoff et al., 2010a; Andtbacka et al., 2015). There is also a promising cancer vaccine PROSTVAC, but the ultimate outcome from phase III clinical trial has proven to be a failure (Gulley et al., 2019).

### Sipuleucel-T

Sipuleucel-T is an infusional autologous DC vaccine, generated by incubating patient’s peripheral blood mononuclear cells (PBMCs) with the recombinant protein PA2024, composed of prostate acid phosphatase (PAP) fused to granulocyte–macrophage colony-stimulating factor (GM-CSF), which was FDA approved for asymptomatic or minimally symptomatic mCRPC in 2010 (Kantoff et al., 2010b). Immunological analysis demonstrated an increase in PAP-specific T cells and activated lymphocytes recruitment into the tumor microenvironment (TME) following vaccination (Fong et al., 2014). Remarkably, sipuleucel-T also elicits humoral immune response to nontargeted tumor antigens, known as antigen cascade and associated with improved clinical outcomes (Guha et al., 2015).

There are three randomized phase III trials to evaluate the safety and efficacy of sipuleucel-T. The pivotal IMPACT trial enrolled 512 patients randomized (2:1) to receive sipuleucel-T or placebo administered three intravenous infusions at 2-week intervals. The study demonstrated a 4.1-month median survival improvement (25.8 vs. 21.7 months) and an extended 3-year survival (31.7% vs. 23.0%) in sipuleucel-T group compared with placebo (Kantoff et al., 2010a). Common adverse events (AEs) included chills (54.1%), pyrexia (29.3%), headache (16%), and influenza-like illness (9.8%), primarily occurring within 1 to 2 days after infusion. Most AEs were mild to moderate (grades 1–2), and no treatment-related autoimmune complications were reported. The integrated analysis of two other clinical trials (D9901 and D9902A) showed a relative reduction of 33% in the risk of death for sipuleucel-T arm compared to placebo (Higano et al., 2009). However, sipuleucel-T vaccination did not prolong the time to disease progression and induce survival benefit without tumor shrinkage or prostate-specific antigen (PSA) declines (Kantoff et al., 2010b).

### T-VEC

T-VEC is an intralesional oncolytic viral vaccine created by genetically engineered herpes simplex virus type 1, in which partial viral genes (ICP34.5 and ICP47) are deleted and replaced by a gene encoding GM-CSF (Liu et al., 2003). The modified virus infects both cancerous and healthy cells but only selectively replicate within tumors, causing the cells to swell and finally be lysed to release tumor-associated antigens (TAAs) (Kohlhapp and Kaufman, 2015). Meanwhile, vaccine viruses also utilize the translation mechanism of cancer cells to secrete GM-CSF, attracting DCs to the TME and stimulating them to present TAA (Toda et al., 2000). In 2015, T-VEC was approved by FDA for the treatment of unresectable nodal, cutaneous, and subcutaneous lesions in recurrent melanoma.

In a phase III OPTiM study, patients (n = 436) with unresected stages III to IV melanoma were randomly assigned (2:1) to intralesional T-VEC or subcutaneous GM-CSF. Compared to GM-CSF, T-VEC significantly improved DRR (16.3% vs. 2.1%) and overall response rate (26.4% vs. 5.7%) and resulted in a trend toward prolonged median OS (23.3 vs. 18.9 months; *P* = 0.051) (Andtbacka et al., 2015). The subgroup of patients with stages IIIB to IVM1a melanoma or treatment-naive disease achieved greater benefit from T-VEC. The most common AEs were fatigue (50%), chills (49%), pyrexia (43%), nausea (36%), and flu-like symptoms (30%). Only grades 3 to 4 AE in ≥2% of patients was cellulitis (2.1%), but no fatal treatment-related AEs occurred (Andtbacka et al., 2015). Furthermore, T-VEC also conduced a complete resolution in 22% of uninjected nonvisceral lesions, as well as 9% of visceral lesions, suggesting that it can generate systemic antitumor immunity to induce tumor regression distant from injection site (Andtbacka et al., 2016).

### PROSTVAC

PROSTVAC (PSA-TRICOM) is a recombinant poxviral vectors vaccine, composed of heterologous prime-boost regimen: the vaccinia priming vaccine and the fowlpox boosting vaccine, which contains human PSA as encoded antigen and a triad of immune costimulatory molecules designated TRICOM: B7.1 (CD80), LFA-3 (CD58), and ICAM-1 (CD54) (Madan et al., 2009). In a previous phase II randomized trial, PROSTVAC prolonged median OS by 8.5 months (25.1 vs. 16.6 months) and improved 3-year survival (30% vs. 17%) in mCRPC compared with placebo (Kantoff et al., 2010a). Unfortunately, in the subsequent larger phase III study, no effective treatment had activities on primary endpoint—median OS; Criteria for futility were met, and ultimately the trial was terminated early (Gulley et al., 2019). Most frequently reported AEs were injection-site reactions (62%) as expected; common non–injection site events and cardiac-related events were fatigue (21%) and arrhythmias (1.4%), respectively. The majority of AEs (>75%) were mild (grade 1), and all serious treatment-related AEs occurred in less than 1% of patients. PROSTVAC is capable of increasing tumor-infiltrating lymphocytes (TILs) and generating specific immune responses against PSA and cascade antigens (Gulley et al., 2014). Combination therapy of PROSTVAC and ICI is currently being investigated in other clinical trials.

## Rationale for Combination Immunotherapy

Growing preclinical data and clinical trials have shown that combination therapy of vaccines and ICIs can trigger intensified immunogenicity and also improve immunosuppressive TME, increasing efficacy than either treatment alone (Pardoll, 2012; Karyampudi et al., 2014). Here, we provide a brief summary of the rationale for combination immunotherapy.

### Intensified Immunogenicity

Several studies have shown that ICI therapy alone has impressive activity in tumors with previous tumor-infiltrating immune response, for example, non–small cell lung cancer (NSCLC) and melanoma (Brahmer et al., 2015; Robert et al., 2015). However, ICIs are regretfully ineffective in nonimmunogenic tumors such as prostatic and pancreatic cancers for the lack of underlying immune recognition (Royal et al., 2010; Beer et al., 2016). Cancer vaccines can generate tumor-specific T cells in periphery or *in situ* tumors and are capable of driving these activated peripheral T cells into the TME leading to increased TILs (Fong et al., 2014; Gulley et al., 2014). Moreover, vaccine-mediated tumor cell death leads to the release of more cascade antigens and induces stronger immune responses specific to antigens not contained within the vaccine, a phenomenon referred to as antigen cascade or epitope spreading (Gregor et al., 2004; Guha et al., 2015). Thus, the hypothesis was proposed that greater efficacy of ICI treatment may be achieved by optimizing tumor immunogenicity or host immune responses with vaccines.

### Improved Immunosuppressive TME

A major challenge for cancer vaccines is that despite the activation of tumor-specific immune responses, immunosuppressive TME restricts effector T-cell function (Thompson et al., 2007; Ahmadzadeh et al., 2009). CTLA-4 is mainly expressed on T helper cells and regulatory T cells (Tregs), mediating inhibitory effects during antigen presentation in periphery by interaction with ligands CD80 or CD84 (Baxter and Hodgkin, 2002; Pardoll, 2012). CTLA-4 inhibitors can directly block these negative signalings to enhance vaccine-induced tumor-specific T cells. CTLA-4 blockade also impacts on Tregs to increase the proportion of effector T cells to Tregs in the TME, which shifts intratumoral balance from immune suppression toward permissive status (Quezada et al., 2006; Liakou et al., 2008). PD-1 plays a critical inhibitory role in modulating the proliferation and cytolytic function of tumor-specific T cells *via* interaction with the ligand PD-L1. Blockade of PD-1 can prevent the senescence of vaccine-activated T cells in the TME, thereby prolonging antitumor activity of effector T cells and can restore the down-regulation of cytokine (interleukin 2, interferon γ [IFN-γ], and tumor necrosis factor α) to promote the cytotoxic effects (Wang et al., 2009; Postow et al., 2015). Taken together, ICIs may enhance and maintain vaccine-induced immune responses by favorably altering immunosuppressive TME and blocking these negative regulations.

## Cancer Vaccines and ICI Combinations

Based on above considerations, a host of clinical trials have been completed or are currently underway. Although many combination studies are in early phases, most of them support the concept of synergy that combining ICIs and therapeutic cancer vaccines has the potential to improve clinical outcomes.

### Combining Anti–CTLA-4 and Vaccines

#### Ipilimumab Plus T-VEC

The phase II trial evaluated ipilimumab combined with T-VEC versus ipilimumab alone for unresectable stage IIIB to IV melanoma patients (n = 198). T-VEC was given intratumorally at first dose ≤4 ml × 10^6^ pfu/ml, after 3 weeks at subsequent doses ≤4 ml × 10^8^ pfu/ml every 2 weeks; ipilimumab 3 mg/kg was intravenously administered every 3 weeks for up to four doses (Puzanov et al., 2016). The objective response rate (ORR) was significantly higher in combination therapy than ipilimumab alone (39% vs. 18%). Moreover, 52% of patients treated with the combination and 23% of patients who received ipilimumab alone had a decrease in uninjected visceral lesions. Frequently occurring AEs for the combination were fatigue (59%), chills (53%), diarrhea (42%), pruritus (40%), and rash (39%), and incidence rates of grade ≥3 AEs in the combination and ipilimumab alone were 45% and 35%, respectively. Three patients with combination therapy had fatal AEs, but none were treatment related (Chesney et al., 2018). These data indicated that the combination had enhanced antitumor activity without additional toxicity compared to ipilimumab alone.

#### Ipilimumab Plus Sipuleucel-T

A small phase I trial of sipuleucel-T in combination with dose-escalation ipilimumab included nine men with docetaxel-naive progressive mCRPC. Subjects received three doses of sipuleucel-T every 2 weeks, immediately followed by low-dose ipilimumab 1 mg/kg given intravenously for a total of one, two, or three doses every 3 weeks (Scholz et al., 2017). Three patients died of disease progression. For six survivors, the median survival has surpassed 50.5 months compared with 35 months in phase III trials of enzalutamide or abiraterone. Tumor-specific antibodies directed at PAP and PA2024 demonstrated a significant increase after sipuleucel-T vaccination and a further elevation after ipilimumab treatment (Ku et al., 2018). There was no unexpected toxicity from combination therapy, and AEs of sipuleucel-T were consistent with previous reports. Ipilimumab led to only a transient grade 1 rash and resolved without additional treatment. Promising survival data and immunological properties in this study support further clinical trials of the combination in larger patient populations and higher doses of ipilimumab.

#### Ipilimumab Plus PROSTVAC

The phase I trial assessed dose-escalation ipilimumab combined with fixed-dose PROSTVAC for patients (n = 30) with mCRPC. PROSTVAC was subcutaneously given at prime doses of 2 × 10^8^ pfu/ml, with subsequent monthly at boost doses of 1 × 10^9^ pfu/ml. Intravenous ipilimumab was administered at doses of 1, 3, 5, and 10 mg/kg on the same day as vaccine. Median OS with the combination in all dose cohorts was 31.3 months, and for patients receiving ipilimumab 10 mg/kg, it was 37.2 months, remarkably longer than historical controls of PROSTVAC or ipilimumab alone (Singh et al., 2015). In total, 58% (14/24) of chemotherapy-naive patients had PSA declines from baseline, and 25% of them had PSA decreases of more than 50% (Jochems et al., 2014). The combination did not exacerbate irAEs associated with ipilimumab, and no dose-limiting toxicity (DLT) was recorded. Grades 1 to 2 injection-site reactions were most common AEs, and rash was frequently reported irAEs mostly occurred in patients treated with ipilimumab 10 mg/kg. Grades 3 to 4 irAEs were observed in eight patients (27%), including rash, diarrhea, colitis, and endocrine events, requiring replacement hormones or supportive measures (Madan et al., 2012). These findings are particularly notable, given that ipilimumab alone has yet to show clinical benefit in mCRPC.

#### Ipilimumab Plus GVAX

GVAX is an engineered cellular vaccine derived from allogeneic cancer cells transfected with GM-CSF, which has been shown to induce durable and specific antitumor immune responses (Lutz et al., 2011). A phase I trial of fixed-dose GVAX plus dose-escalation ipilimumab was conducted in chemotherapy-naive mCRPC. All patients (n = 28) received GVAX intradermally at a priming dose of 5 × 10^8^ cells with subsequent injections at a dose of 3 × 10^8^ cells every 2 weeks for 24 weeks and intravenous ipilimumab at extended doses of 0.3, 1, 3, and 5 mg/kg every 4 weeks. The study demonstrated >50% PSA declines from baseline in 25% (7/28) of patients, and four patients obtained stable disease measured by bone scan (Gerritsen et al., 2008). Most common AEs (>30%) were grades 1 to 2 injection-site reactions, fatigue, fever, influenza-like symptoms, and rash. At 5 mg/kg dose level, one patient underwent grade 4 sarcoid alveolitis defined as DLT. Other grade 3 irAEs included hypophysitis and hepatitis, both related to ipilimumab and responding to hormone replacement therapy (Eertwegh et al., 2012). Overall, irAEs with the combination appeared to be manageable.

Another phase Ib trial evaluated ipilimumab with or without GVAX in previously treated advanced pancreatic adenocarcinoma. Patients (n = 30) were randomized (1:1) to receive intravenous ipilimumab 10 mg/kg alone or intradermal GVAX at doses of 5 × 10^8^ cells with subsequent ipilimumab 10 mg/kg. Compared with ipilimumab alone, the combination had prolonged disease stabilization (31, 71, and 81 weeks for three patients vs. 7 and 22 weeks for two patients), improved 1-year survival (27% vs. 7%), and a trend of favorable median OS (5.7 vs. 3.6 months; *P* = 0.072) (Le et al., 2013). CA19-9 biochemical responses were observed in 47% (7/15) of patients with combination therapy, whereas none in ipilimumab alone. Most common AEs in combination therapy were grades 1 to 2 injection-site reactions, rash, fatigue, fever, and influenza-like illness. Similar to previous ipilimumab reports, 20% of patients experienced grades 3 to 4 irAEs including rash, colitis, pneumonitis, and nephritis. All irAEs responded to steroids with the exception of nephritis requiring hemodialysis (Le et al., 2013). Further researches on the combination of ICIs and GVAX in the treatment of mCRPC or pancreatic cancer are warranted.

#### Ipilimumab Plus Peptide Vaccine

The efficacy of ipilimumab plus peptide vaccination (gp100) was explored in progressive stage IV melanoma patients (n = 56), who received two different doses of ipilimumab concomitantly with gp100 vaccination. The study demonstrated a durable objective response correlating with autoimmunity and tumor regression (Attia et al., 2005). Unfortunately, in pivotal phase III study for previously treated advanced melanoma, ipilimumab combined with gp100 was negative. Patients (n = 676) were randomly assigned (3:1:1) to ipilimumab plus vaccine, ipilimumab alone, or vaccine alone. Gp100 emulsified with incomplete Freund’s adjuvant (IFA) was subcutaneously injected, and ipilimumab 3 mg/kg was given intravenously every 3 weeks for up to 3 months. No difference in median OS was detected between the combination and ipilimumab alone (10 vs. 10.1 months; *P* = 0.76). The best ORR was 10.9% in ipilimumab alone compared to 5.7% in combination arm (Hodi et al., 2010). The irAEs were similar in ipilimumab with or without vaccine, which most often affected skin and gastrointestinal tract. Although four patients required infliximab for grades 3 to 4 diarrhea or colitis, most of irAEs are reversible with corticosteroids or hormone replacement therapy. Ultimately, these data did not indicate any improved clinical outcome of ipilimumab plus peptide vaccine.

Other studies evaluated ipilimumab combined with peptide vaccines (MART-1/gp100/tyrosinase with Montanide ISA 51 VG) as adjuvant setting in high-risk resected stages IIIC to IV melanoma. In first single-arm trial, patients (n = 19) received three different doses of ipilimumab with multipeptide. The study showed that response rate to specific peptides (47%) was higher than previous reports, and disease relapse rate was lower in patients with autoimmunity (Sanderson et al., 2005). Subsequently, another phase II trial enrolled 75 patients randomized (2:1) to receive extended-dose ipilimumab (3 or 10 mg/kg) every 6 to 8 weeks, along with subcutaneous immunizations of peptide vaccines. Although activated T cells increased over time after vaccination, only 25% of patients had immune responses to specific multipeptide. Autoimmune evidence positively correlating with improved relapse-free survival (RFS) was observed in 37% of patients, but the combination failed to generate additional benefits (Sarnaik et al., 2011). The AEs with the combination are generally reversible, and there were no treatment-related deaths. Frequently occurring grades 3 to 4 AEs were diarrhea, colitis, and hypopituitarism, which occurred in 29% of patients. All required tapering doses of systemic steroids, and most patients returned to normal within 3 months. In brief, adjuvant ipilimumab plus peptide vaccine following resection of high-risk melanoma had no impressive clinical activity.

### Combining Anti–PD-1/PD-L1 and Vaccines

#### Pembrolizumab Plus T-VEC

The phase Ib trial evaluated pembrolizumab plus T-VEC for the treatment of unresectable stages IIIB to IV melanoma. Patients (n = 21) received T-VEC at initial dose of 4 ml × 10^6^ pfu/ml, followed 3 weeks later at full dose of 4 ml × 10^8^ pfu/ml every 2 weeks. Pembrolizumab 200 mg was administered intravenously coinciding with subsequent doses of T-VEC (Long et al., 2015). The confirmed ORR was 62%, about twice as shown in phase III study of pembrolizumab (34%) and T-VEC (26%), and complete response rate for per immune-related response criteria was 33%. An increase in lymphocytes infiltration, PD-L1 protein, and IFN-γ gene expression was observed in patients responded to combination therapy. The combination did not increase toxicity of monotherapy, with fatigue (62%), chills (48%), fever (43%), rash (33%), and arthralgia (33%) as the most common AEs. Only one grade 1 AEs associated with the combination resulted in hospitalization, while other grades 3 to 4 AEs were solely due to pembrolizumab (Ribas et al., 2017). Subsequently, the further phase III KEYNOTE-034 trial of systemic administration of pembrolizumab with intralesional injection of T-VEC is ongoing (NCT02263508).

Similarly, the phase Ib study evaluated pembrolizumab combined with T-VEC in patients (n = 36) with advanced squamous cell carcinoma of the head and neck. T-VEC was injected intralesionally at first dose of 8 ml × 10^6^ pfu/ml, then at subsequent doses of 8 ml × 10^8^ pfu/ml every 3 weeks. Intravenous pembrolizumab 200 mg was administered every 3 weeks (Harrington et al., 2017). Preliminary data from this study showed that the ORR was 16.7% (six patients with five subjects PD-L1 positive), and disease control rate was 38.9% (14 patients with 11 subjects PD-L1 positive). The most common AEs for the combination were pyrexia (36.1%), dyspnea (33.3%), and fatigue (25.0%). Grades 3 to 4 AEs were observed in 24 patients (66.7%), of which two (5.6%) and one (2.8%) patients discontinued treatment attributed to T-VEC and pembrolizumab, respectively. In one patient, DLT occurred: fatal arterial hemorrhage (Harrington et al., 2018). But overall, combination therapy was considered to have manageable safety, with amended protocol to exclude patients who received the neck reirradiation or at high risk of arterial hemorrhage (Harrington et al., 2017).

#### Nivolumab Plus Peptide Vaccine

In the phase I trial, therapeutic efficacy of nivolumab with or without multipeptide vaccines was assessed in ipilimumab-refractory and -naive melanoma. Patients (n = 90) with unresectable stages III to IV melanoma were treated with extended dose of nivolumab (1, 3, or 10 mg/kg) with or without peptide vaccines (MART-1/NY-ESO-1/gp100 with Montanide ISA 51 VG) (Kudchadkar et al., 2012). For both ipilimumab-refractory and -naive subjects, the RECIST response rates were 25%, and nivolumab-induced durable responses for up to 140 weeks. Combination therapy was well tolerated and safe, and no treatment-related death occurred. The common AEs were fatigue and injection-site reaction, most of which were mild to moderate and easy to manage. Other grade 3 irAEs (optic neuritis, fever, pneumonitis, and rash) can be resolved by prednisone taper as described previously for nivolumab. However, immunoassay demonstrated no increased responses in patients’ PBMC to multipeptide at all doses and finally confirmed that peptide vaccines failed to improve clinical efficacy of nivolumab (Weber et al., 2013).

The same group conducted the phase I trial of nivolumab plus multipeptide vaccines as adjuvant setting in resected stages IIIC to IV melanoma. Patients (n = 33) were treated with extended dose of nivolumab (1, 3, or 10 mg/kg) plus peptide vaccines (MART-1/NY-ESO-1/gp100 with Montanide ISA 51 VG) every 2 weeks for 24 weeks, followed by nivolumab alone every 3 months for up to 2 years (Gibney et al., 2015). Estimated median RFS was 47.1 months, extremely beneficial compared with historical median RFS (12–21 months) (Hsueh et al., 2002; Sosman et al., 2011). The median OS was not reached with median follow-up of 32.1 months, and relapse rate at that time significantly decreased to 30.3%. Most common AEs (>40%) were injection-site reaction, fatigue, rash, pruritus, nausea, and arthralgia. Treatment-related grade 3 AEs included hypokalemia, rash, enteritis, and colitis, and only one toxicity meeting the DLT criteria was colitis. All related AEs responded to systemic management of steroids and supportive care (Gibney et al., 2015). This study suggested that nivolumab plus peptide vaccines can produce immunologic activity and promising survival as adjuvant therapy for high-risk advanced melanoma.

### Emerging Progress in Combination Strategy

Lately, the combination of ICIs with antigen-presenting cell administration, especially DC vaccines, has been explored as an encouraging therapeutic strategy. The phase II study investigated ipilimumab combined with TriMixDC-MEL, created by autologous DCs electroporated with synthetic mRNA, in pretreated advanced melanoma (Wilgenhof et al., 2016). Patients (n = 39) were administered TriMixDC-MEL subcutaneously and intravenously plus ipilimumab 10 mg/kg every 3 weeks for four doses, followed by nivolumab maintenance every 3 months. The disease control rate was 51% at 6 months, and ORR with the combination was 38%, which was higher than ipilimumab monotherapy (10%–15%). Tumor responses included eight complete and seven partial responses, half of which are ongoing after median follow-up of 3 years. The most common AEs (>30%) consisted of injection-site reactions, influenza-like illness, dermatitis, and chills, and no treatment-related deaths occurred. A total of 14 patients (36%) underwent grades 3 to 4 events, but most AEs were reversible by using established treatment algorithms (Wilgenhof et al., 2016).

Other studies undertook ICIs combined with intratumoral injection of innate immune activators, particularly Toll-like receptor 9 (TLR9) agonist, as a potential approach to improve clinical benefits. The phase I trial of tremelimumab plus subcutaneous administration of TLR9 agonist (CPG 7909) in stage IV melanoma or other advanced solid tumors demonstrated durable (>170 days) partial responses in 12% (2/17) of the patients with good tolerability (Millward et al., 2013). Another phase Ib study evaluated pembrolizumab plus intratumoral SD-101, a synthetic CpG oligonucleotide as TLR9-stimulating factor, in unresectable or metastatic melanoma. Among nine anti–PD-1 therapy-naive patients, the ORR was 78%, and 1-year progression-free survival rate was 88%. Combination therapy induced increased TILs in the TME and durable tumor responses in uninjected visceral lesions. SD-101 vaccination most often led to transient grades 1 to 2 injection-site reactions and influenza-like illness, and combination therapy had minimal additional toxicity relative to pembrolizumab alone (Ribas et al., 2018). Likewise, at the 2018 American Association for Cancer Research Annual Meeting, preliminary data from phase Ib trial of pembrolizumab plus intratumoral TLR9 agonist CMP-001, a CpG-A oligodeoxynucleotide packaged in virus-like particles, demonstrated a remarkable improvement in ORR of 33% for advanced melanoma previously resistant to anti–PD-1 therapy (Milhem et al., 2018).

## Challenges and Future Perspectives

### Management of Combination-Related AEs

Recently, dual checkpoint blockade (combining ipilimumab and nivolumab) has demonstrated improved response rates in advanced melanoma and NSCLC; however, the benefit comes with drawbacks of additional toxicity (Antonia et al., 2015; Larkin et al., 2015). In contrast, observed toxicity with the combination of ICIs and cancer vaccines was within previously described spectrum of AEs for monotherapy ICI or vaccine, and no novel-toxicity was reported. Vaccination most often led to mild to moderate (grade 1 or 2) injection-site reactions, pyrexia, fatigue, and flu-like symptoms, appearing as transient symptoms at the early stage (Kantoff et al., 2010b; Andtbacka et al., 2015). Clinical toxicity related to ICIs covers a series of tissue-specific inflammatory events known as irAEs, which affect but are not limited to skin (rash, pruritus), gastrointestinal (diarrhea, colitis), endocrine (thyroiditis, hypophysitis), lung (pneumonitis), kidney (nephritis), and liver (hepatitis) (Weber et al., 2012; Kyi and Postow, 2016). Although severe irAEs may result in prolonged hospitalizations and even fatalities, the frequency of grade 3 or higher irAEs for combination therapy does not increase compared with ICI or vaccine alone.

The management of combination-related AEs is similar to that of immunotherapy alone, the majority of irAEs with the combination of ICIs and cancer vaccines are reversible when treatment is discontinued and/or managed with standard immunosuppressive algorithms such as steroids, and on occasion infliximab for refractory diarrhea or hepatitis (Weber et al., 2012; Champiat et al., 2015; Kyi and Postow, 2016). Details about management strategies of specific irAEs have been comprehensively reviewed (Champiat et al., 2015; Puzanov et al., 2017), and we highlight the importance of early recognition and prompt intervention. The median remission time for endocrine-related toxicity is longer, requiring continued but not necessarily permanent hormone replacement therapy; long-term effects of combination therapy and whether different ranges of irAEs will exhibit during chronic exposure have yet to be observed (Weber et al., 2012; Kyi and Postow, 2016). Additionally, irAEs are dose dependent and appear to be correlated with improved median OS, but are not a prerequisite for therapeutic efficacy. These results are based on retrospective analysis of small samples and so warrant further clinical exploration (Eertwegh et al., 2012; Owen et al., 2017).

### Optimization of Vaccine Platforms

Despite the limited efficacy of vaccine monotherapy, cancer vaccines as key components of combination therapy can generate tumor-specific immune responses associated with survival (Sheikh et al., 2013). There are several key considerations for vaccine design needed to be emphasized. Accumulating evidence (e.g., the failure of gp100 peptide) indicated that immune responses elicited by peptide vaccines may be transient or of low magnitude and insufficient to enhance the efficacy of ICIs (Slingluff, 2011; Hirayama and Nishimura, 2016), while peptide-loaded autologous DC vaccines with strong immunogenicity and well tolerance have demonstrated remarkable clinical activities when combined with ICIs (Gatti-Mays et al., 2017). Besides, vaccines emulsified with IFA may lead to tumor-specific T-cell sequestration, dysfunction, and eventually apoptosis at injection site instead of destroying tumors, which is another major cause of peptide–IFA vaccine failure (Hailemichael et al., 2013). Thus, combination strategies are being optimized by applying suitable vaccine preparations and adjuvants, for example, DCs, viral vectors, or TLR agonists acting on innate immunity (Hailemichael et al., 2018).

**Figure 1:**
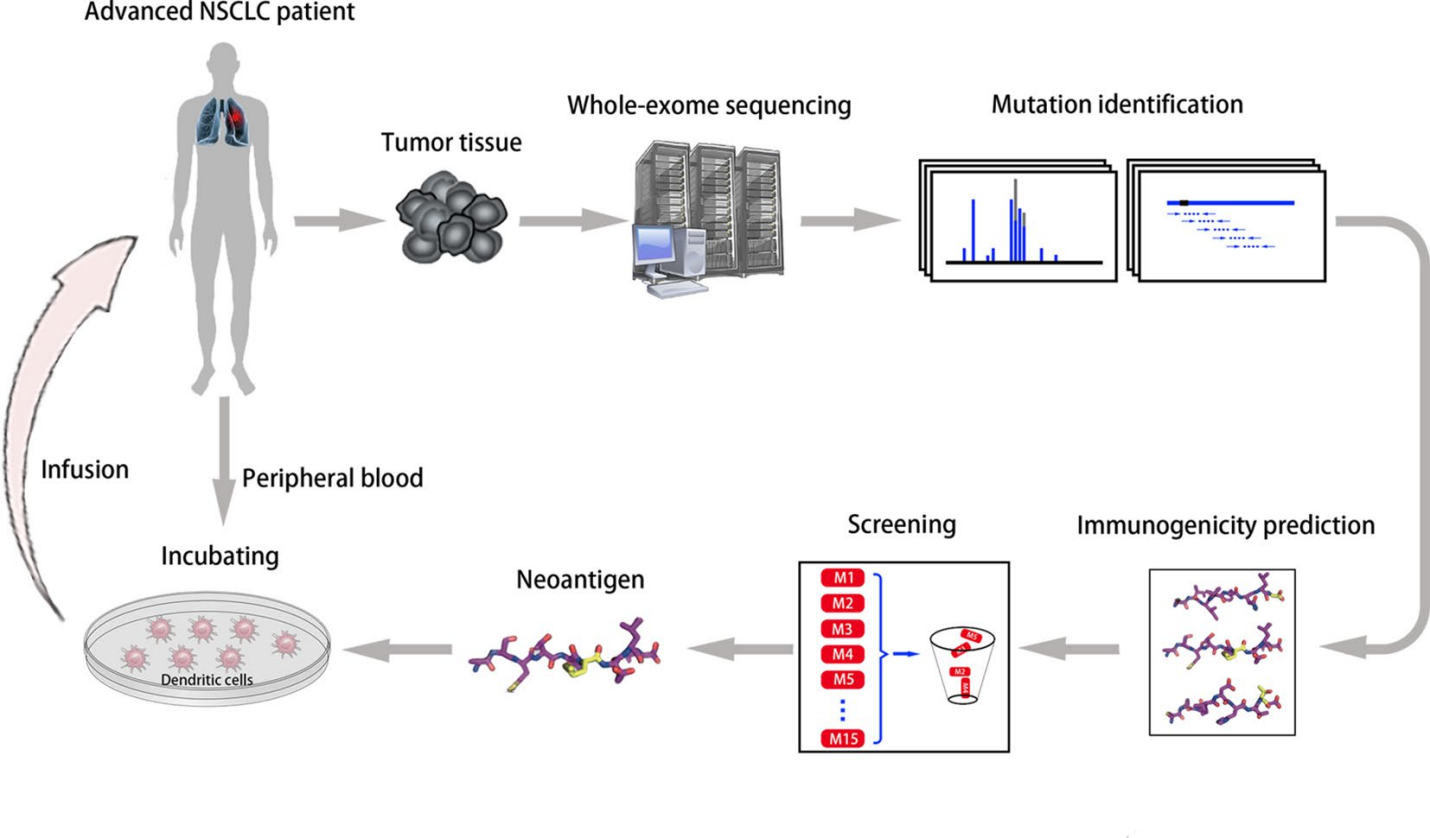
Neoantigen-primed personalized DC vaccines for refractory NSCLC. We utilized whole-exome sequencing of tumor tissues, computational epitope prediction, and immunological approaches to screen for neoantigens from individual patients, and then infused autologous DCs pulsed with neoantigen-derived peptides into each patient (ClinicalTrials.gov Identifier: NCT02956551).

Another vital factor in vaccine design is the selection of antigen targets. The majority of identified tumor antigens are self-antigens with lower affinity for TCR molecules inducing less robust clinical responses, and targeting these antigens may result in increased toxicity (Cloosen et al., 2007; Collins et al., 2018). Conversely, neoantigens derived from somatic mutations with minimal central immune tolerance and theoretical limited toxicity have become an optimal strategy for vaccine development (Stone et al., 2015; Hirayama and Nishimura, 2016). We conducted a phase I trial of neoantigen-primed DC vaccines for individualized treatment of refractory NSCLC (NCT02956551, **Figure 1**). As of May 2019, the study enrolled 11 patients, eight of whom finally received vaccination. Preliminary data demonstrated good tolerance, with only one patient developing a rash. Seven patients obtained stable disease with median progression-free survival of 5.7 months (range, 3.8–10.0 months) (Ding et al., 2019). Notably, two independent small-scale phase I studies of neoantigen-targeted personalized vaccines showed that three patients received vaccination plus ICIs, and all experienced complete tumor regression (Ott et al., 2017; Sahin et al., 2017). These findings indicate that “precise target” tumor vaccines combined with ICIs will become a priority candidate for antitumor therapy.

### Time Sequence and Clinical Settings

Different combination strategies of vaccines and ICIs may have dissimilar ideal schedule. Checkpoint receptors change after vaccination in a time-dependent manner; namely, CTLA-4 expression decreased significantly 7 days after T-cell activation, whereas PD-1 expression persistently increased for a longer period (Fend et al., 2017). Studies showed that CTLA-4 blockade restrained tumor growth most availably when administered 1 day after vaccination, while administration on the same day did not produce antitumor activities. Anti–PD-1 treatment was most effective when administered 7 days after vaccination (Rojas et al., 2015; Fend et al., 2017). In another research, anti–CTLA-4 administration on the day of vaccination, or 1 day after instead of before, can maximize intratumoral CD8^+^ T cell infiltration and tumor-specific lysis (Wada et al., 2013). However, other evidence indicated that administration of CTLA-4 and PD-1 blockade prior to vaccination still reduced tumor progression and improved long-term survival (Espenschied et al., 2003; Ali et al., 2016). Preclinical studies on time sequence of combination therapy are yet to entirely consistent, and predicting their manner of translation in clinical settings is difficult.

Furthermore, preclinical studies showed that combining vaccines and ICIs did not improve survival in prophylactic murine model (immunization before tumor inoculation), but did extend survival in therapeutic model, may be owing to epitope spreading caused by immunogenic cell death after initial vaccination (Davila et al., 2003; Williams et al., 2013). As above, combination therapy appeared to improve clinical outcomes in adjuvant postoperative therapy. Patients had preexisting tumors and often for years or may remain microscopic metastases after surgery, which provided antigens to prime underlying immune responses (Gibney et al., 2015; Morse and Lyerly, 2015). The Cancer Vaccine Consortium recommended the introduction of therapeutic cancer vaccines in early-stage and/or low-volume disease, but fortunately, combination therapy with ICIs may extend the scope of vaccine application to advanced or metastatic clinical settings (Finke et al., 2007; Dillman, 2017).

### Biomarkers for Combination Therapy

The selection of appropriate patient population for immunotherapy is all important, but to date, no effective predictive biomarkers have been found. Consistent data suggest that PD-L1 expression alone is insufficient to predict response to immunotherapy, that is, negative PD-L1 staining does not preclude the response (Weber et al., 2013; Shen and Zhao, 2018). Besides, the expression of PD-L1 in the TME is dynamic adaptive changes, while detection of PD-L1 expression in pretreatment biopsy only provides single static assessments (Sawada et al., 2015; Boussiotis, 2016). Recent studies showed that mismatch repair deficiency and high mutational burden may generate neoantigens and increase tumoral immunogenicity, which have become new biomarkers for response to ICI treatment (Snyder et al., 2014; Le et al., 2017).

However, the value of predictive biomarkers may observably change with combination therapy of vaccines and ICIs. Immunological analysis of nivolumab plus vaccines demonstrated a remarkable increase in peripheral Tregs and decrease in antigen-specific T cells in nonresponders and those with progressive disease (Weber et al., 2013). In adjuvant setting, a trend toward lower baseline peripheral Tregs and myeloid-derived suppressor cells was observed in nonrelapsing patients, and PD-L1 expression was not associated with RFS (Gibney et al., 2015). Similarly, in the study of ipilimumab combined with vaccine, the frequency of Tregs increased in patients with progressive disease, resulting in a shorter survival (Santegoets et al., 2013). Significantly improved OS was seen in patients with pretreatment high levels of CD4^+^CTLA-4^+^, CD4^+^PD-1^+^, and differentiated CD8^+^ T cells or low levels of Tregs and differentiated CD4^+^ T cells (Santegoets et al., 2013). All these findings highly implicated that depletion of Tregs may be one of the key factors to enhance therapeutic efficacy of the combination.

## Conclusion

Cancer vaccines monotherapy produce only modest clinical benefits, but as key components of combination therapy, they can generate tumor-specific immune responses associated with survival. Many combination studies are in early phases, most of which support that combining ICIs and cancer vaccines holds maximized potential to improve clinical outcomes. Importantly, the combination has minimal additional toxicity compared to single-agent vaccines or ICIs. Personalized cancer vaccines have become a priority option for vaccine design, and potential strategies of combining these “precise target” vaccines with ICIs lack full testing but hold great promise. Moreover, the selection of appropriate patient population for immunotherapy is all important, but to date, no single immunology or tumor characteristic is sufficient to predict response to combination therapy and warrants further study.

## Author Contributions

J-YL and JZ contributed conception and overall idea of the study; JZ wrote the first draft of the manuscript; YC and Z-YD wrote sections of the manuscript. All authors contributed to manuscript revision, read, and approved the submitted version.

## Funding

This work was partly supported by the National Natural Science Foundation of China (no. 81572380), National Clinical Research Center for Geriatrics (West China Hospital, Z2018B12), and 1.3.5 Project for Disciplines of Excellence, West China Hospital, Sichuan University (ZYJC18022).

## Conflict of Interest

The authors declare that the research was conducted in the absence of any commercial or financial relationships that could be construed as a potential conflict of interest.
